# Platelet Distribution Width at First Day of Hospital Admission in Patients with Hemorrhagic Fever with Renal Syndrome Caused by Hantaan Virus May Predict Disease Severity and Critical Patients' Survival

**DOI:** 10.1155/2018/9701619

**Published:** 2018-06-19

**Authors:** Xiude Fan, Zitong Liu, Shiqi Fu, Jiao Sang, Huan Deng, Fang Li, Xiaoge Zhang, Na Li, Qunying Han, Zhengwen Liu

**Affiliations:** ^1^Department of Infectious Diseases, First Affiliated Hospital of Xi'an Jiaotong University, Xi'an, 710061 Shaanxi, China; ^2^Xi'an Medical University, Xi'an, 710021 Shaanxi, China

## Abstract

Thrombocytopenia is one of the main characteristics of hemorrhagic fever with renal syndrome (HFRS). This study aimed to evaluate the associations of platelet distribution width (PDW) with the disease severity and critical patients' survival of HFRS. The demographics, clinical data, and white blood cell and platelet parameters including PDW in 260 patients hospitalized for HFRS were analyzed. The results showed that PDW on the first day (PDW1) was positively associated with the disease severity (*p* = 0.005). Multiple regression analysis showed that in addition to age (odds ratio [OR], 1.091; 95% confidence interval [CI], 1.015–1.172) and occurrence of sepsis (OR, 22.283; 95% CI, 2.985–166.325), PDW1 (OR, 0.782; 95% CI, 0.617–0.992) was a risk factor of the mortality, having an area under the receiver operating characteristic curve of 0.709 (95% CI, 0.572–0.846, *p* = 0.013) for predicting mortality, with a sensitivity of 70% and a specificity of 67% at a cutoff of 16.5 fL, in patients with critical HFRS. These results suggest the potential of PDW at the first day of hospitalization as a valuable parameter for evaluating the severity of HFRS and a moderate parameter for predicting the prognosis of critical HFRS patients. A prospective study in large patient population is needed to validate these findings.

## 1. Introduction

Hemorrhagic fever with renal syndrome (HFRS), a zoonotic disease caused by pathogenic hantaviruses, is characterized by altered vascular permeability all over the body and may exhibit severe profile with sudden fever, acute renal failure, shock, and hemorrhage [1]. HFRS is mainly endemic in Asia, Southeast Europe, and North Africa [2–4]. China is the most seriously affected country and accounts for over 90% of the total number of HFRS cases all over the world. In China, two HFRS-causing hantaviruses, Hantaan and Seoul viruses, which induce a severe and a mild form of HFRS, respectively, are the main agents of HFRS diseases and the endemic of these two viruses is geographically quite stable [5]. Due to the promotion of vaccine in China, the prevalence of HFRS has been decreased in recent years [6]. However, there has been no specific antiviral therapy for HFRS up to now and the high mortality of critical cases persists in certain areas [7]. According to the criteria of clinical classification of China, HFRS is classified into four clinical types: mild, medium, severe, and gravis [8]. These criteria of clinical classification depend largely on doctors' subjective judgment, and the incidence of atypical patients with unusual clinical manifestations has increased. Currently, no reliable prognostic parameters have been available for the patients, especially the critically ill patients. These may hamper the early diagnosis and treatment of HFRS. Therefore, exploring new and early predictors to assess the severity and prognosis of HFRS beforehand is important to guide the clinician to initiate effective treatment and improve clinical management.

Platelets (PLTs), fragments of megakaryocyte cytoplasm, play a prothrombotic role in the process of stopping bleeding at the site of the interrupted endothelium [9]. Platelet parameters obtained by automated cell counters consist of PLT count, platelet distribution width (PDW), mean platelet volume (MPV), platelet-large cell rate (P-LCR), and plateletcrit (PCT). Decreased PLT count was shown to precede acute kidney failure in patients with Puumala virus infections [10], and PLT count was an independent predictor of severe HFRS [11] and critically ill patients in intensive care unit (ICU) [12]. In addition, PDW, MPV, P-LCR, and PCT are associated with platelet activation and function and have recently been shown to be useful markers for predicting occurrence and prognosis of certain inflammatory diseases and vascular diseases, such as Crimean-Congo hemorrhagic fever [13], sepsis [14], pulmonary embolism [15], ascitic fluid infection [16], obstructive sleep apnea [17], Crohn's disease [18], and coronary artery disease [19]. It is well known that platelet parameters are routinely tested on HFRS patients in hospitals almost every day. Nevertheless, there is limited information on their clinical significance in HFRS.

The aim of the current study, therefore, was to investigate whether platelet parameters would be useful in evaluating the severity of HFRS disease and predicting the prognosis of HFRS patients.

## 2. Materials and Methods

### 2.1. Study Population

This retrospective study was conducted in patients from the First Affiliated Hospital of Xi'an Jiaotong University. The patients were all residents of Shaanxi Province in China, an endemic area of Hantaan virus infection. Records of 321 HFRS patients (mean age 41.79 ± 16.75 years; 243 men and 78 women) from January 2011 to December 2016 were collected.

The diagnosis of HFRS was confirmed by clinical manifestations in combination with serological evidence of the presence of IgM or IgM and IgG antibodies against hantavirus determined by indirect immunofluorescence test. For the present investigation, only adult patients were included and patients who had pregnancy, cancer, hemopathy, other kidney diseases, liver diseases, and the use of anticoagulants prior to admission were excluded. Based upon the diagnosis criteria for the prevention and treatment strategy of HFRS published by the Ministry of Health, China, the disease severity in HFRS patients was classified into mild, medium, severe, and gravis types. Accordingly, 58 patients were classified as mild type, 75 medium type, 44 severe type, and 83 gravis type. The patients with gravis type HFRS were divided into nonsurvival and survival groups to analyze factors associated with prognosis.

### 2.2. Data Management

Personal features and clinical data collected from the patient's medical chart included the patient's age, sex, max temperature prior to admission, days from fever onset to hospital admission, blood pressure at the time of the assessment, cigarette and alcohol consumption, hospital stay, blood transfusion, continuous renal replacement therapy (CRRT), comorbidities (hypertension, diabetes mellitus, and coronary heart disease), and HFRS-related complications (hemorrhage, infection, hepatic injury, kidney rupture, sepsis, multiple organ dysfunction syndrome (MODS), and arrhythmia). Hemorrhage refers to patients presenting with bleeding (pulmonary hemorrhage, melena, hematemesis, hematuria, diffuse ecchymosis, and hematoma) throughout hospitalization. Sepsis was defined according to criteria determined by Vincent et al. [19]. Blood transfusion, CRRT, comorbidity, and HFRS-related complications were reported as a total count.

Blood tests routinely measured by autoanalyzers on the day of hospital admission and on the third day of hospitalization were collected. Variables were as follows: WBC, PLT, PDW, MPV, P-LCR, and PCT. Routine blood test results like PDW values on the day of hospital admission were recorded as PDW1 and those on the third day of admission as PDW3. The study protocol was performed to conform with the Declaration of Helsinki and was approved by the Ethics Committee of the First Affiliated Hospital of Xi'an Jiaotong University. Informed consent was not obtained from the patients as all patient records/information was anonymized and deidentified prior to analysis.

### 2.3. Statistical Analysis

Statistical analysis was performed using SPSS 20.0 software (SPSS Inc., Chicago, IL, USA) and MedCalc software version 12 (MedCalc Software, Ostend, Belgium). Quantitative variables were expressed as mean and standard deviation or median and interquartile range, where appropriate. The normality of the distribution and the homogeneity of variance were assessed with the Kolmogorov-Smirnoff test and Levene's test, respectively. Demographic information and blood routine parameters were compared by the independent sample *t*-test or one-way ANOVA test for normally distributed variables. The nonnormally distributed variables were compared by the nonparametric Kruskal-Wallis test. The distributions of MPV levels in the first day conformed to normal distribution, and one-way ANOVA test was used for statistical analysis. Numerical variables such as age, max temperature, blood pressure, hospital stay, and other laboratory parameters did not coincide with normal distribution; these variables were compared by the nonparametric Kruskal-Wallis test. Chi-square test and Fisher exact test were used for categorical variables. Spearman correlation was performed to indicate the direction of association between parameter and disease severity (positive or negative association). Tolerance and variance inflation factor (VIF) were used to detect multicollinearity among variables. Variables significantly associated with death were tested in a logistic regression model for their potential to predict the corresponding outcome. Variables with statistically significant results in the univariate analyses and without multicollinearity were included in multivariate logistic regression analyses for independent variables. Predictive values of the laboratory parameters for prognosis were tested with receiver operating characteristic curves (ROC) and quantified by calculating the area under the ROC curve (AUC) and the 95% confidence interval (CI). MedCalc software was used to test the statistical significance of the difference between the AUCs. A *p* value < 0.05 was considered statistically significant. All *p* values were from two-sided tests.

## 3. Results

### 3.1. Patient Characteristics

According to inclusion and exclusion criteria, records of 260 adult HFRS patients were finally included in this study (Supplementary Figure 1). The characteristics of patients included are shown in Table 1. The 260 patients had a mean age of 44.82 ± 14.99 years, and 74.61% were male (Table 1). Nineteen HFRS patients died, with a mortality rate of 5.91%. As expected, HFRS patients with the gravis type had significantly higher mortality rate (16/83, 19.3%) than patients with mild (0/58, 0%), medium (2/75, 2.7%), and severe (1/44, 2.3%) types. There were no significant differences in age, sex, max temperature, blood pressure, consumption of tobacco and alcohol, or comorbidities between patients with different severity of the disease.

### 3.2. Complication Related to the Severity of HFRS Patients

Among HFRS-related complications, the incidences of hemorrhage, infection, and hepatic injury increased gradually as the severity of the disease increased (Table 1). Kidney rupture, sepsis, and MODS were only observed in severe and gravis patients. Arrhythmia was not identified in mild patients. Furthermore, the severe and gravis patients tended to have longer hospital stays and more frequent blood transfusions and CRRT than the mild and medium patients (*p* < 0.001, Table 1).

### 3.3. Laboratory Parameters Related to the Severity of HFRS Patients

The levels of WBC count, PLT count, PDW, MPV, P-LCR, and PCT on the first day of admission demonstrated significant differences among the four clinical types. Levels of WBC1 and WBC3 increased gradually with the severity of the disease. On the first day of admission, PDW and MPV levels in the severe and gravis patients were higher than those in the mild and medium patients. Levels of PLT and PCT in the first and third day were decreased significantly with the severity of the disease, and these two indices in the mild or moderate patients were higher than those in the severity and gravis patients. The ratios of day 1 parameters of hospital admission to those of day 3 parameters of admission were not significantly different among the four clinical types except the ratio of WBC1 to WBC3 level. WBC1/WBC3 ratios in the medium, severe, and gravis patients were higher than those in the mild patients (Table 2).

Spearman correlation analysis further confirmed that WBC1, WBC3, WBC1/WBC3, and PDW levels were positively correlated with the severity of disease (all *p* < 0.05), while the levels of PLT1, PLT3, PCT1, and PCT3 were negatively correlated with the severity of HFRS (all *p* < 0.05; Supplementary Table 1).

### 3.4. Factors Associated with the Mortality of Patients with Gravis HFRS

Since most of the deaths occurred in the patients with gravis HFRS among the four clinical types (16/19, 84.21%), factors associated with mortality of patients with gravis HFRS were analyzed. Many variables including age, systolic (SBP) and diastolic blood pressure (DBP), and incidences of HFRS-related complications were significantly associated with deaths of HFRS patients (Table 3). Age was significantly higher in the nonsurvivors than that in the survivors. In contrast, lower SBP and DBP was common in those who died compared to those who survived. Among HFRS-related complications, the incidences of secondary infection, sepsis, and MODS in the nonsurvivors were significantly higher than those of the survivors. No significant differences in the frequencies of hemorrhage, hepatic injury, and arrhythmia were identified (Supplementary Table 2). Shorter hospital stays were found in the nonsurvivors compared to survivors due to the death of gravis patients during the disease course.

A comparison of platelet indices between survivor and nonsurvivor groups is given in Supplementary Table 3. On the first day of admission, PDW and MPV levels were significantly higher in the survivor group than those in the nonsurvivor group (*p* = 0.024 and *p* = 0.04, resp.). There was no significant difference in the third day PDW (*p* = 0.488) and MPV levels (*p* = 0.962) between both groups. PDW1/PDW3 in those who died was 1.096 ± 0.26 compared to 0.92 ± 0.16 in survivors (*p* = 0.035; Supplementary Table 3).

Multicollinearity test indicated no significant multicollinearity (all tolerance values > 2.8 and all VIF values < 3.5). Multiple logistic regression analysis showed that age (OR, 1.091; 95% CI, 1.015–1.172; *p* = 0.018), PDW levels at the first day of hospital admission (OR, 0.782; 95% CI, 0.617–0.992; *p* = 0.043), and sepsis secondary to HFRS (OR, 22.283; 95% CI, 2.985–166.325; *p* = 0.002) were independent risk factors for the mortality in HFRS patients after adjustment for MPV1, PDW1/PDW3, SBP, DBP, secondary infection, and MODS (Table 3).

**Figure 1:**
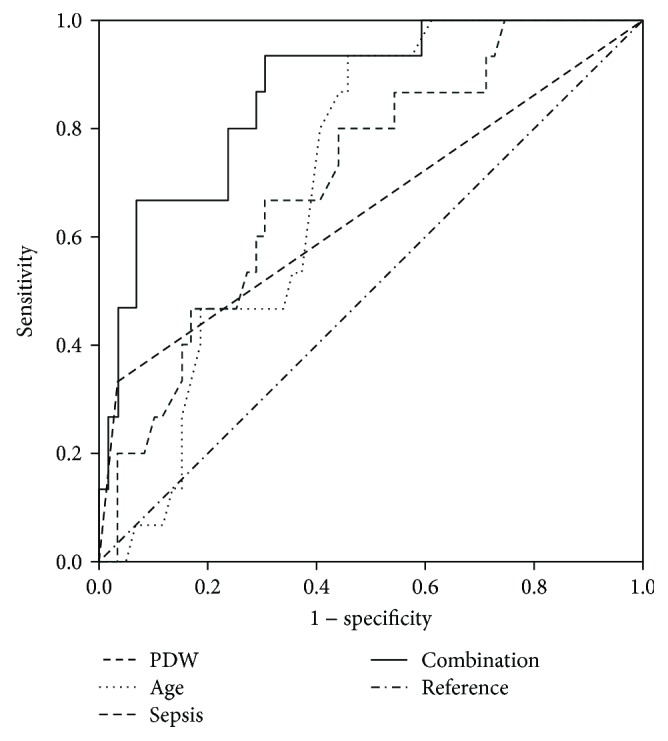
Receiver operating characteristic curve (ROC) analysis of platelet distribution width at first day of hospital admission (PDW1), age, and sepsis and the combination of PDW1, age, and sepsis to predict prognosis in critical HFRS patients.

To explore the predictive value of age, PDW1 levels, and the incidences of sepsis on the prognosis of HFRS, ROC analysis was performed. PDW1 was the platelet indices showing a high AUC (0.709), with a sensitivity of 70% and a specificity of 67% at a cutoff of 16.5 fL. All pertinent data are shown in Table 4. ROC analysis demonstrated that the specificity, sensitivity, and AUC of the three factors in combination were preferable to any single factor alone in predicting prognosis (*p* < 0.05; Table 4, Figure 1).

## 4. Discussion

The current study was performed to investigate the predictive role of the routine blood parameters of WBC and PLT in the setting of HFRS. We analyzed the demographic, epidemiological, clinical and laboratory characteristics of 260 patients who were hospitalized due to HFRS. The main findings are that WBC and PLT counts in the first and third day of hospital admission, WBC1/WBC3, PDW1, and PCT3, were correlated with the severity of HFRS. The patients' age, reduced PDW1, and the incidence of sepsis were significant predictors of HFRS mortality in critical patients.

Platelets, small anucleate cell fragments of the megakaryocytes, play a key role in regulating haemostasis and protecting vascular integrity [8, 20]. Platelet indices including PLT, PDW, MPV, P-LCR, and PCT are standard indicators of platelet function. PDW is an indicator of the heterogeneity in platelet size, which can be a sign of active platelet release. MPV represents the average size of platelets in the blood. An increase in PDW and MPV suggests an increased range of platelet size and increased average diameter of the platelets, respectively, due to swelling, destruction, and immaturity [12, 13, 15–21]. PCT is the ratio of total platelets in the blood. A reduction of PCT and PLT simultaneously indicates that platelets have been consumed or destructed [22]. P-LCR is more sensitive to changes in platelet size and is often correlated to MPV. Just like with MPV and PDW, P-LCR is inversely related to the platelet counts and is useful for the differentiation of thrombocytopenia [23].

HFRS is an acute viral disease caused by a pathogenic hantavirus and has the clinical characteristics of thrombocytopenia and systemic inflammatory response syndrome [24]. Our study showed that compared with patients of mild and medium types of HFRS, patients with severe and gravis types tend to require more frequent blood transfusions and CRRT and suffer more fatal complications such as hemorrhage, hepatic injury, kidney rupture, sepsis, and MODS. Furthermore, the nonsurvivors had higher incidences of secondary infection, sepsis, and MODS.

So far, the pathogenesis of HFRS is not fully known. The virus itself, immune response, and interaction between the host immune system and hantaviruses may contribute to disease initiation and development. Vascular endothelial dysfunction is the basic pathological change, which is characterized by a dramatic increase in vascular permeability [25]. Currently, there is no specific therapy for HFRS. Early diagnosis and prognosis is important for the management of the patients. Therefore, early identification of patients with HFRS at risk of developing severe and gravis types would potentially improve the management via prompt initiation of systematic supportive treatment.

Platelet parameters have been suggested as valuable novel biomarkers. Activation of the coagulation system [26, 27], severe infection [13–15, 21, 25], inflammatory diseases [18], trauma [28], cardiovascular diseases [19], and thrombotic diseases [29] could all result in changes in platelet parameters. Previous study showed that PLT counts were negatively correlated with the progression of HFRS, and the AUC values were 0.814 for the severity of HFRS, which indicated the better predictive efficacy of PLT counts [11]. However, the patients in that study [11] were just divided into mild group and severe group, and no analysis of the predictive value of platelet parameters was conducted in HFRS patients in relation to the patients' survival.

It is generally recognized that when PLTs have been excessively destructed and consumed, bone marrow will produce a mass of immature PLTs which have larger volume than mature ones. At that time, PDW, MPV, and P-LCR will be increased correspondingly, because immature platelets with large volume and mature platelets with small volume synchronously were present in the blood [30]. Because thrombocytopenia is a significant feature of HFRS, the analysis of all the platelet parameters, instead of only PLT count, may provide a more accurate and comprehensive information for the disease severity and patient prognosis.

We analyzed whether WBC count, PLT count, PDW, MPV, P-LCR, or PCT could predict disease severity and mortality in HFRS. Firstly, levels of WBC and platelet parameters were investigated and analyzed in relation to clinical types of HFRS. Consistent with previous studies [11, 31], WBC count on day 1 of hospital admission (WBC1) was found to increase gradually with the exacerbation of the disease severity. Patients with mild type HFRS had higher PLT1 value than those with severe and gravis types. Of the 4 platelet parameters routinely tested, levels of MPV1 and PDW1 in the severe and gravis patients were found to be higher than those in the mild and medium patients. PCT1 was consistent with the variation trend of PLT1. Secondly, this study examined factors associated with patient survival in patients with gravis HFRS. PDW1 was found to be a significant predictor of mortality of patients with gravis type HFRS.

In contrast to the increasing trend of PDW1 with disease severity among the four clinical types of HFRS, PDW1 was decreased in nonsurvivors compared with survivors. This might suggest that the depletive reduction of platelets cannot be compensated by the hemopoietic function due to this critical disease condition. However, the possible mechanism for the decrease of PDW1 in nonsurvivors of patients with gravis type HFRS is not very clear and needs further investigations.

It is not difficult to understand that age and the occurrences of sepsis were also found to be independent risk factors of death in gravis HFRS patients.

Studies on risk scores in patients with other diseases showed that laboratory parameters at day 3 and the ratio of the first-day value to the third-day value had better predictive performance than baseline data [13, 32, 33]. One could hypothesize that the variation of platelet parameters within the first 3 days after hospital admission could be a strong predictor of clinical deterioration and mortality. Therefore, we compared the prediction accuracy of WBC and platelet parameters at day 3 and ratios of WBC and platelet parameters at day 1 to WBC and platelet parameters at day 3, respectively. Although WBC3, PLT3, PCT3, and WBC1/WBC3 were found to be correlated with the severity of HFRS, these parameters were not superior to parameters at day 1 in the prediction accuracy. Therefore, waiting 3 or more days seems to confer no benefit to improving the prediction performance of platelet parameters.

Our study has a few limitations. First, the clinical classification criteria of HFRS contain substantial subjective factors based on symptoms and signs, which may influence the judgment of severity. Second, the study is limited by the relatively small number of critical patients, especially for the binary logistic regression analysis used for identifying risk factors for prognosis. Third, the study is a retrospective design and has no validation analysis in other patient populations. Fourth, this study shows that PDW1 may only moderately predict mortality and thus caution should be exercised when using this marker. Prospective studies with a larger sample of HFRS patients are needed to verify the predictive value of these parameters in the severity and mortality of HFRS patients.

## 5. Conclusion

The current study showed that WBC, PLT, PDW, and PCT may be used as valuable parameters for the severity of HFRS patients. Furthermore, in gravis HFRS patients, reduced PDW1, age, and the occurrences of sepsis were risk factors for prognosis and the combination of these factors had better performance than any single parameter alone in predicting the mortality of gravis HFRS patients. Platelet distribution width at the first day of hospital admission (PDW1) has the potential as a valuable parameter for the disease severity of HFRS and a moderate parameter for the prognosis of critical HFRS patients in that its alteration is a significant factor associated with the disease severity of HFRS and the survival of gravis HFRS patients. The early determination of PDW and the dynamic and close monitoring of sepsis occurrence may help clinicians to identify high-risk HFRS patients and to stratify risk for optimal management.

## Figures and Tables

**Table 1:** Demographic and clinical characteristics of the patients with HFRS of different clinical types.

Variables	Mild (*n* = 58)	Medium (*n* = 75)	Severe (*n* = 44)	Gravis (*n* = 83)	*p* value
Male, *n* (%)	39 (67.2)	53 (70.7)	36 (81.8)	66 (79.5)	0.209
Age, years	43 (30.5)	42 (23)	48. 5(27.25)	49 (20)	0.105
Max temperature, °C	38.8 (1.42)	39 (1.60)	39 (1.27)	39 (1.3)	0.140
Admitted days after fever, days	5 (3.00)	5 (3.00)	6 (2.00)	5 (3.00)	0.070
SBP, mmHg	120 (17.25)	121 (23)	125 (29.5)	117 (19)	0.058
DBP, mmHg	76 (14)	77 (15)	83.5 (20.50)	77 (16)	0.177
Smoking, *n* (%)	20 (34.5)	30 (40)	24 (54.5)	40 (48.2)	0.158
Alcohol consumption, *n* (%)	21 (36.2)	25 (33.3)	21 (47.7)	37 (44.6)	0.314
Comorbidity					
Hypertension, *n* (%)	7 (12.1)	9 (12)	10 (22.7)	13 (15.7)	0.390
Diabetes mellitus, *n* (%)	3 (5.2)	3 (4)	0	2 (2.4%)	0.285
Coronary heart disease, *n* (%)	2 (3.4)	2 (2.7)	2 (4.5)	3 (3.6)	0.457
Number of deaths, *n* (%)	0	2 (2.7)	1 (2.3)	16 (19.3)	<0.001
HFRS-related complication					
Hemorrhage, *n* (%)	22 (37.90)	32 (42.70)	30 (68.20)	62 (74.70)	<0.001
Secondary infection, *n* (%)	23 (39.70)	34 (45.30)	19 (43.20)	49 (59.00)	0.098
Hepatic injury, *n* (%)	25 (43.10)	40 (53.30)	23 (52.30)	61 (73.50)	0.013
Sepsis, *n* (%)	0	0	1 (2.30)	7 (8.40)	0.003
MODS, *n* (%)	0	0	0	11	<0.001
Arrhythmia, *n* (%)	0	5 (6.7)	5 (11.40)	6 (7.2)	0.026
Kidney rupture, *n* (%)	0	0	1 (2.30)	1 (1.2)	0.260
Hospital stay, days	10 (6)	10 (4)	13 (8.50)	15 (13)	<0.001
Blood transfusion, *n* (%)	7 (12.10)	12 (16)	24 (54.50)	56 (67.50)	<0.001
CRRT, *n* (%)	0	0	16 (36.40)	58 (69.90)	<0.001

SBP: systolic blood pressure; DBP: diastolic blood pressure; MODS: multiple organ dysfunction syndrome; CRRT: continuous renal replacement therapy.

**Table 2:** Routine blood parameters in HFRS patients of different clinical types.

Parameters	Mild (*n* = 58)	Medium (*n* = 75)	Severe (*n* = 44)	Gravis (*n* = 83)	*p* value
WBC1, ×10^9^ cells/L	7.35 (3.97)	9.2 (7.73)	10.1 (6.16)	13.07 (12.18)	<0.001
PLT1, ×10^9^ cells/L	100 (96.5)	61 (68)	51 (48.50)	41 (47)	<0.001
PDW1 (fL)	16.0 (3.50)	16.6 (6.10)	17.8 (4.90)	17.15 (4.45)	0.003
MPV1 (fL)	11.88 ± 1.49	12.47 ± 1.30	12.77 ± 1.15	12.46 ± 1.52	0.017
P-LCR1 (%)	39.7 (16.25)	44.10 (13.40)	45.9 (6.10)	43.2 (11.35)	0.020
PCT1 (%)	0.12 (0.10)	0.09 (0.12)	0.07 (0.07)	0.06 (0.07)	<0.001
WBC3, ×10^9^ cells/L	7.16 (5.22)	9.49 (4.99)	8.41 (4.79)	10.27 (7.76)	<0.001
PLT3, ×10^9^ cells/L	115.5 (159.25)	125 (145.25)	77 (112)	60 (67)	<0.001
PDW3 (fL)	16.2 (2.80)	16.2 (5.02)	16.3 (2.35)	16.8 (3.65)	0.391
MPV3 (fL)	12.1 (2.72)	12.4 (2.05)	12.3 (1.40)	12.4 (1.63)	0.213
P-LCR3 (%)	41 (20)	44.2 (15.67)	42.8 (8)	42.45 (11.5)	0.482
PCT3 (%)	0.17 (0.12)	0.16 (0.16)	0.12 (0.13)	0.09 (0.09)	<0.001
WBC1/WBC3	1 (0.6)	1.06 (0.53)	1.20 (0.63)	1.16 (0.69)	0.005
PLT1/PLT3	0.78 (0.43)	0.64 (0.54)	0.55 (0.41)	0.70 (0.68)	0.082
PDW1/PLW3	1 (0.25)	1.01 (0.26)	1.14 (0.27)	1.01 (0.33)	0.083
MPV1/MPV3	1 (0.11)	1.01 (0.11)	1.03 (0.13)	1.02 (0.17)	0.717
P-LCR1/P-LCR3	1 (0.21)	1.03 (0.24)	1.05 (0.26)	1.03 (0.31)	0.540
PCT1/PCT3	0.78 (0.40)	0.71 (0.53)	0.64 (0.53)	0.71 (0.74)	0.187

WBC: white blood cell; PLT: platelet; PDW: platelet distribution width; MPV: mean platelet volume; P-LCR: platelet-large cell rate; PCT: plateletcrit.

**Table 3:** Independent risk factors associated with death in patients with gravis HFRS.

Variables	Univariable logistic regression		Multivariable logistic regression	
	OR (95% CI)	*p* value	OR (95% CI)	*p* value
Age	1.060 (1.009, 1.114)	0.019	1.091 (1.015, 1.172)	0.018
SBP	0.981 (0.948, 1.015)	0.264		
DBP	0.970 (0.935, 1.006)	0.097		
Secondary infection	6.400 (1.349, 30.372)	0.019		
MODS	12.250 (2.981, 50.333)	0.001		
Sepsis	14.773 (2.542, 85.859)	0.003	22.283 (2.985, 166.325)	0.002
PLT1	0.997 (0.988, 1.005)	0.431		
PLT3	0.995 (0.984, 1.005)	0.297		
PDW1	0.818 (0.679, 0.985)	0.034	0.782 (0.617, 0.992)	0.043
PDW3	1.086 (0.863, 1.368)	0.483		
MPV1	0.658 (0.435, 0.995)	0.047		
MPV3	0.987 (0.592, 1.647)	0.962		
PDW1/PDW3	0.05 (0.003, 0.899)	0.042		

OR: odds ratio; CI: confidence interval; SBP: systolic blood pressure; DBP: diastolic blood pressure; MODS: multiple organ dysfunction syndrome; PLT: platelet; PDW: platelet distribution width; MPV: mean platelet volume.

**Table 4:** Predictive values of platelet parameters for prognosis of patients with gravis HFRS.

Variables	AUC	*p* value	Cutoff value	Sensitivity	Specificity	95% CI for AUC	
						Lower	Upper
PDW1	0.709	0.013	16.5	0.70	0.67	0.572	0.846
Age	0.707	0.014	45.5	0.94	0.48	0.588	0.826
Sepsis	0.650	0.075	—	—	—	0.474	0.825
Combination	0.867	<0.001	0.12^**a**^	0.93	0.70	0.770	0.963

AUC: area under the receiver operating characteristic (ROC) curve; CI: confidence interval; PDW1: platelet distribution width at the first day of hospital admission. ^**a**^Probability value of the combination was analyzed by logistic regression. The regression coefficients of these three parameters were used to set up a logit model of death for critical HFRS patients as follows: Logit(P|*y* = death) = −5.322 + 3.104 × sepsis − 0.246 × PDW + 0.087 × age.

## Data Availability

The data underlying the findings of this study are all included in this article and its supplementary materials.

## Supplementary Materials

Supplementary 1 Table 1: correlation of laboratory data and the severity of HFRS.

Supplementary 2 Table 2: demographic and clinical characteristics of survivors and nonsurvivors in patients with gravis HFRS.

Supplementary 3 Table 3: comparison of white blood cell count and platelet parameters between survivors and nonsurvivors in patients with gravis HFRS.

Supplementary 4 Figure 1: flowchart of the patients' selection process in the study.
