# Supplementary material for: Platelet Distribution Width at First Day of Hospital Admission in Patients with Hemorrhagic Fever with Renal Syndrome Caused by Hantaan Virus May Predict Disease Severity and Critical Patients' Survival
Source: Dis Markers. 2018 Jun 19;2018:9701619. doi: 10.1155/2018/9701619 (PMC6029476; doi:10.1155/2018/9701619)
Supplement: Supplementary 1 — Table 1: correlation of laboratory data and the severity of HFRS. [file 9701619.f1.doc]

**Supplementary Table 1: Correlation of laboratory data and** the severity of HFRS.

| **Parameters** | **r** | ***p* value** |
| --- | --- | --- |
| **WBC1** | 0.412 | <0.001 |
| **PLT1** | -0.363 | <0.001 |
| **PDW1** | 0.184 | 0.005 |
| **PCT1** | -0.324 | <0.001 |
| **WBC3** | 0.258 | <0.001 |
| **PLT3** | -0.336 | <0.001 |
| **PCT3** | -0.312 | <0.001 |
| **WBC1/WBC3** | 0.201 | 0.001 |

**r**, correlation coefficient.
