# Supplementary material for: Platelet Distribution Width at First Day of Hospital Admission in Patients with Hemorrhagic Fever with Renal Syndrome Caused by Hantaan Virus May Predict Disease Severity and Critical Patients' Survival
Source: Dis Markers. 2018 Jun 19;2018:9701619. doi: 10.1155/2018/9701619 (PMC6029476; doi:10.1155/2018/9701619)
Supplement: Supplementary 2 — Table 2: demographic and clinical characteristics of survivors and nonsurvivors in patients with gravis HFRS. [file 9701619.f2.doc]

**Supplementary Table 2: Demographicand clinical characteristics of survivors and non-survivors in patients with gravis HFRS.**

| **Variables** | **Survivors (n = 67)** | **Non-survivors (n = 16)** | ***p* value** |
| --- | --- | --- | --- |
| **Male, n (%)** | 55(82.10) | 11(68.80) | 0.235 |
| **Age, years** | 47(19) | 58(11) | <0.001 |
| **Hospital stay, days** | 18.63±8.70 | 7.81±6.23 | <0.001 |
| **Max temperature, °C** | 38.97±0.85 | 38.94±0.72 | 0.910 |
| **Admitted Days after fever** | 5.86±3.87 | 6.37±2.91 | 0.624 |
| **SBP, mmHg** | 120(23) | 114(23) | <0.001 |
| **DBP, mmHg** | 80(16) | 72(11) | <0.001 |
| **Smoking, n (%)** | 31(46.30) | 9(56.2) | 0.473 |
| **Alcohol consumption, n (%)** | 29(43.30) | 8(50) | 0.627 |
| **Comorbidity** |  |  |  |
| Hypertension, n (%) | 8(11.90) | 5(31.20) | 0.127 |
| Diabetes mellitusm, n (%) | 1(1.50) | 1(6.20) | 0.350 |
| Coronary heart disease, n (%) | 2(3) | 1(6.20) | 0.479 |
| **HFRS-related complication** |  |  |  |
| Hemorrhage, n (%) | 47(70.10) | 15(93.80) | 0.103 |
| Secondary infection, n (%) | 35(52.20) | 14(87.50) | 0.010 |
| Hepatic injury, n (%) | 48(71.60) | 13(81.20) | 0.640 |
| MODS, n (%) | 4(6) | 7(43.8) | <0.001 |
| Sepsis, n (%) | 2(3) | 5(31.2) | 0.002 |
| Kidney rupture, n (%) | 1(1.50) | 0 | P>0.05 |
| Arrhythmia, n (%) | 5 (7.50) | 1(6.20) | P>0.05 |
| **Blood transfusion, n (%)** | 45(67.20) | 11(68.80) | 0.903 |
| **CRRT, n (%)** | 44(65.70) | 14(67.50) | 0.160 |

SBP, systolic blood pressure; DBP, diastolic blood pressure; MODS, multiple organ disfunction syndrome; CRRT, continuous renal replacement therapy.
