# Supplementary material for: Platelet Distribution Width at First Day of Hospital Admission in Patients with Hemorrhagic Fever with Renal Syndrome Caused by Hantaan Virus May Predict Disease Severity and Critical Patients' Survival
Source: Dis Markers. 2018 Jun 19;2018:9701619. doi: 10.1155/2018/9701619 (PMC6029476; doi:10.1155/2018/9701619)
Supplement: Supplementary 3 — Table 3: comparison of white blood cell count and platelet parameters between survivors and nonsurvivors in patients with gravis HFRS. [file 9701619.f3.doc]

**Supplementary Table 3: Comparison of white blood cell count and platelet parameters between survivors and non-survivors in patients with gravis HFRS.**

| **Parameters** | **Survivors (n = 67)** | **Non-survivors (n = 16)** | ***p* value** |
| --- | --- | --- | --- |
| **WBC1, ×10^9^ cells/L** | 15.7±8.74 | 17.9±17.49 | 0.452 |
| **PLT1, ×10^9^ cells/L** | 74.56±97.37 | 54.50±44.69 | 0.425 |
| **PDW1, (fL)** | 17.97±3.76 | 15.62±2.37 | 0.024 |
| **MPV1, (fL)** | 12.65±1.53 | 11.74±1.31 | 0.040 |
| **P-LCR1, (%)** | 52.67±62.87 | 40.81±8.72 | 0.471 |
| **PCT1, (%)** | 0.10±0.10 | 0.06±0.05 | 0.148 |
| **WBC3, ×10^9^ cells/L** | 11.61(7.72) | 9.95(13.45) | 0.848 |
| **PLT3, ×10^9^ cells/L** | 90.77±88.52 | 65.13±55.51 | 0.287 |
| **PDW3, (fL)** | 16.73±2.60 | 17.30±2.72 | 0.488 |
| **MPV3 , (fL)** | 12.37±1.15 | 12.36±1.34 | 0.962 |
| **P-LCR3, (%)** | 43.42±8.08 | 43.23±7.87 | 0.938 |
| **PCT3, (%)** | 0.12±0.10 | 0.08±0.06 | 0.221 |
| **WBC1/WBC3,** | 1.37±0.79 | 1.15±0.41 | 0.312 |
| **PLT1/PLT3** | 1.11±1.40 | 0.97±0.46 | 0.719 |
| **PDW1/PDW3** | 1.096±0.26 | 0.92±0.16 | 0.035 |
| **MPV1/MPV3** | 1.03±0.15 | 0.96±0.17 | 0.144 |
| **P-LCR1/P-LCR3** | 1.26±1.53 | 0.96±0.25 | 0.481 |
| **PCT1/PCT3** | 1.08±1.19 | 1±0.82 | 0.832 |
