# Supplementary figures and images for: Platelet Distribution Width at First Day of Hospital Admission in Patients with Hemorrhagic Fever with Renal Syndrome Caused by Hantaan Virus May Predict Disease Severity and Critical Patients' Survival
Source: Dis Markers. 2018 Jun 19;2018:9701619. doi: 10.1155/2018/9701619 (PMC6029476; doi:10.1155/2018/9701619)

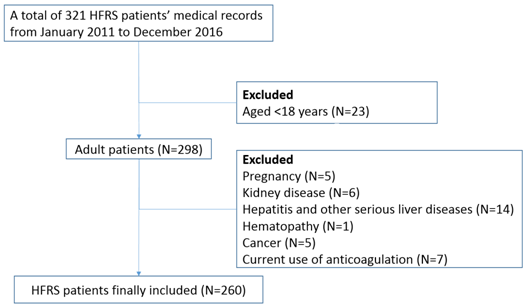

Supplement: Supplementary 4 — Figure 1: flowchart of the patients' selection process in the study. [file 9701619.f4.tif]
